# Suppose We Had No Sugar

**Published:** 1885-01

**Authors:** 


### SUPPOSE WE HAD NO SUGAR.

Besides the natural sweets we have taken to producing artificial ones. Has any housewife ever realized the alarming condition of cookery in the benighted generations before the invention of sugar? It is really almost too appalling to think about. So many things that we now look upon as all but necessaries—cakes, puddings, made dishes, preserves, confectionery, sweet biscuits, jellies, cooked fruits, tarts, &c.—were then practically quite impossible. Fancy attempting nowadays to live a single day without sugar; no tea, no coffee, no jam, no pudding, no cake, no sweets, no hot toddy before one goes to bed; the bare idea of it is too terrible. And yet that was really the abject condition of all the civilized world up to the middle of the Middle Ages. Horace's punch was sugarless and lemonless; the gentle Virgil never tasted the congenial cup of afternoon tea ; and Socrates went from his cradle to his grave without ever knowing tha flavor of peppermint bull'seyes. How the children managed to spend their Saturday *as*, or their weekly *obolus*, is a profound mystery. To be sure, people had honey ; but honey is rare, dear, and scanty ; it can never have filled one-quarter the place that sugar fills in our modern affections. Try for a moment to realize drinking honey with one's whiskey and water, or doing the year's preserving with a pot of the best Narborine, and you get at once a common measure of the difference between the two as practical sweeteners. Nowadays we get sugar from cane and beetroot in abundance, while sugar maples and palm trees of various sorts afford a considerable supply to remoter countries. But the childhood of the little Greeks and Romans must have been absolutely unlightened by a single ray of joy from chocolate creams or Everton toffee. The consequence of this excessive production of sweets in modern times is, of course, that we have begun to distrust the indications afforded us by the sense of taste in this particular as to the wholesomeness of various objects. We can mix sugar with anything we like, whether it had sugar in it to begin with or otherwise, and by sweetening and flavoring we can give a false palatableness to even the worst and most indigestible rubbish, such as plaster of Paris, largely sold under the name of sugared almonds to the ingenuous youth of two hemispheres. But in untouched nature the test rarely or never fails. As long as fruits are unripe and unfit for human food they are green and sour ; as soon as they ripen they become soft and sweet, and usually acquire some bright color as a sort of advertisement of their edibility. In the main, bar the accidents of civilization, whatever is sweet is good to eat—nay more, is meant to be eaten; it is only our own perverse folly that makes us sometimes think all nice things bad for us, and all wholesome things nasty. In a state of nature, the exact opposite is really the case. One may observe, too, that children, who are literally young savages in more senses than one, stand nearer to the primitive feeling in this respect than grown-up people. They unaffectedly like sweets : adults, who have grown more accustomed to the artificial meat diet, don't as a rule, care much for puddings, cakes, and made dishes. (May I venture parenthetically to add, any appearance to the contrary notwithstanding, that I am not a vegetarian, and that I am far from desiring to bring down upon my devoted head the imprecation pronounced against the rash person who would rob a poor man of his beer. It is quite possible to believe that vegetarianism was the starting point of the race, without wishing to consider it also as the goal; just as it is quite possible to regard clothes as purely artificial products of civilization, without desiring personally to return to the charming simplicity of the Garden of Eden.)—*The Cornhill Magazine.*

